# Genetic diversity and population structure of black cottonwood (*Populus deltoides*) revealed using simple sequence repeat markers

**DOI:** 10.1186/s12863-019-0805-1

**Published:** 2020-01-06

**Authors:** Cun Chen, Yanguang Chu, Changjun Ding, Xiaohua Su, Qinjun Huang

**Affiliations:** 1 State Key Laboratory of Tree Genetics and Breeding, Research Institute of Forestry, Chinese Academy of Forestry, Beijing, China; 2 Key Laboratory of Tree Breeding and Cultivation, State Forestry and Grassland Administration, Beijing, China; 3 Co-Innovation Center for Sustainable Forestry in Southern China, Nanjing Forestry University, Nanjing, Jiangsu Province, China

**Keywords:** *Populus deltoides*, Genetic diversity, Population structure, Simple sequence repeat

## Abstract

**Background:**

Black cottonwood (*Populus deltoides*) is one of the keystone forest tree species, and has become the main breeding parents in poplar hybrid breeding. However, the genetic diversity and population structure of the introduced resources are not fully understood.

**Results:**

In the present study, five loci containing null alleles were excluded and 15 pairs of SSR (simple sequence repeat) primers were used to analyze the genetic diversity and population structure of 384 individuals from six provenances (Missouri, Iowa, Washington, Louisiana, and Tennessee (USA), and Quebec in Canada) of *P. deltoides*. Ultimately, 108 alleles (*N*_*a*_) were detected; the expected heterozygosity (*H*_*e*_) per locus ranged from 0.070 to 0.905, and the average polymorphic information content (*PIC*) was 0.535. The provenance ‘Was’ had a relatively low genetic diversity, while ‘Que’, ‘Lou’, and ‘Ten’ provenances had high genetic diversity, with Shannon’s information index (*I*) above 1.0. The mean coefficient of genetic differentiation (*F*_*st*_) and gene flow (*N*_*m*_) were 0.129 and 1.931, respectively. Analysis of molecular variance (AMOVA) showed that 84.88% of the genetic variation originated from individuals. Based on principal coordinate analysis (PCoA) and STRUCTURE cluster analysis, individuals distributed in the Mississippi River Basin were roughly classified as one group, while those distributed in the St. Lawrence River Basin and Columbia River Basin were classified as another group. The cluster analysis based on the population level showed that provenance ‘Iow’ had a small gene flow and high degree of genetic differentiation compared with the other provenances, and was classified into one group. There was a significant relationship between genetic distance and geographical distance.

**Conclusions:**

*P. deltoides* resources have high genetic diversity and there is a moderate level of genetic differentiation among provenances. Geographical isolation and natural conditions may be the main factors causing genetic differences among individuals. Individuals reflecting population genetic information can be selected to build a core germplasm bank. Meanwhile, the results could provide theoretical support for the scientific management and efficient utilization of *P. deltoides* genetic resources, and promote the development of molecular marker-assisted breeding of poplar.

## Background

Genetic diversity is an important component of biodiversity, and is the basis of ecosystem and species diversity [1–3]. Understanding the genetic diversity and structure of germplasm resources aids efficient and rational development, and the utilization of germplasm resources on the premise of effective protection [4–7]. The growth characteristics of trees involve reaching a large size with a long growth cycle; therefore, it is necessary to analyze their genetic diversity and population structure. To fully understand the genetic information of germplasm resources, screening representative individuals and constructing a core germplasm bank for protection and utilization can shorten the breeding process and accelerate genetic improvement [8]. Currently, researchers regard the study of forest genetic diversity and population structure as important basic research, and such studies have been carried out on a variety of species. Among forest trees, the genome of *Populus trichocarpa* was the first to be sequenced [9]; therefore, there have been relatively more studies on its genetic information, including its genetic diversity and population structure [10, 11]. In addition, similar studies have been carried out on other tree species, such as *P. nigra* L. [12–14], *P. simonii* [15], *P. tremuloides* [16], *P. balsamifera* [17], *P. cathayana* Rehd [18], *P. euphratica* [19], *P. tomentosa* [20] and *P. szechuanica var. tibetica* [21].

Many methods and techniques have been developed to advance population genetic diversity research, among which molecular tools play an important role in the management and utilization of genetic resources. In particular, simple sequence repeat (SSR) molecular marker technology is an ideal method because of its simple operation, co-dominance, high resolution, polymorphism, and repeatability [22]. Many species-specific SSR markers have been developed for *P. simonii* [23, 24] and *P. tomentosa* [25–27] by analyzing functional gene sequences, and these markers can be applied to the study of other poplar species.

*P. deltoides* is widely distributed from the Mississippi River to southern Canada in North America, and is one of the keystone forest tree species with important ecological value. This species has been widely used in forest breeding research and its genetic resources have become the main gene donors of poplar cultivars. These cultivars are mainly used to provide feedstocks for pulp, fiber, and bioenergy industries [28]. Furthermore, this tree is currently one of the most suitable for short rotation industrial timber intensive management of woody crops in the mid-latitude areas of the world. Our team collected *P. deltoides* germplasm resources through international exchange and cooperation in 2009, and a germplasm bank of *P. deltoides* was established in China. Production practice showed that the black poplar has a dominant position in the poplar plantation; thus, *P. deltoides* and its hybrid with *P. nigra* L. have become the main commercial poplar trees in China.

Little research has been performed on the genetic diversity and population structure of *P. deltoides*. Fahrenkrog et al. [29] reported the first population genomics study for *P. deltoides* distributed in the Mississippi River Basin to determine its genetic diversity and adaptation potential. In the present study, we analyzed 384 *P. deltoides* individuals preserved in the germplasm bank, which were originally distributed in 27 collection sites for 6 provenances. SSR molecular marker technology was used to study their genetic diversity and population structure. Some of the selected primers were developed based on functional genes sequences, which could be used to study gene function of *P. deltoides.* In this way, more information could be provided for marker-assisted breeding in combination with phenomics. Meanwhile, other (universal) primers were obtained from the International Populus Genome Consortium (IPGC, http://www.ornl.gov/sci/ipgc/ssr_resource.htm) and Washington University (Poplar Molecular Genetics Cooperative, http://poplar2.cfr.washington.edu). Using this strategy, the genetic diversity of the population could be reflected objectively and comprehensively. In addition, based on the results, a core germplasm bank of *P. deltoides* could be built, allowing *P. deltoides* resources to be protected, managed, and utilized more scientifically and rationally.

## Results

### Polymorphic SSR primers

In this study, 20 polymorphic SSR primer pairs were preliminary selected from 145 poplar SSR primer pairs for further analysis (Additional file 1: Table S1). Using two methods to detect the null alleles of the loci (Additional file 1: Table S2) [30, 31], we found that the null alleles of five loci (SSR15, SSR42, SSR54, SSR65, and SSR76) could affect the analysis results; therefore, the data of these five loci were excluded from the subsequent analysis. Finally, 15 SSR primer pairs were selected to analyze the genetic diversity and population structure of *P. deltoides*.

### Microsatellite polymorphisms

A total of 108 alleles were detected using 15 SSR primer pairs among 384 *P. deltoides* individuals. The number of alleles (*N*_*a*_) per locus ranged from 2 (SSR85) to 19 (SSR126), with a mean of 7.2. The average effective number of alleles (*N*_*e*_) per locus was 3.48, ranging from 1.08 at SSR85 to 10.40 at SSR126. Locus SSR126 provided relatively large amounts of genetic information and its Shannon’s information index (*I*) was 2.533. The means of the observed heterozygosity (*H*_*o*_) and expected heterozygosity (*H*_*e*_) were 0.509 and 0.579, respectively. In addition, except for locus SSR104, the false-homozygous phenomenon existed in the other loci (*H*_*o*_ > *H*_*e*_). The polymorphic information content (*PIC*), as one measurement of genetic diversity, was between 0.068 (SSR85) and 0.896 (SSR120), with an average of 0.535. Six loci showed significant deviations from the Hardy-Weinberg equilibrium (HWE), which indicted that there was genetic differentiation among the provenances (Additional file 1: Table S3).

**Fig. 1:**
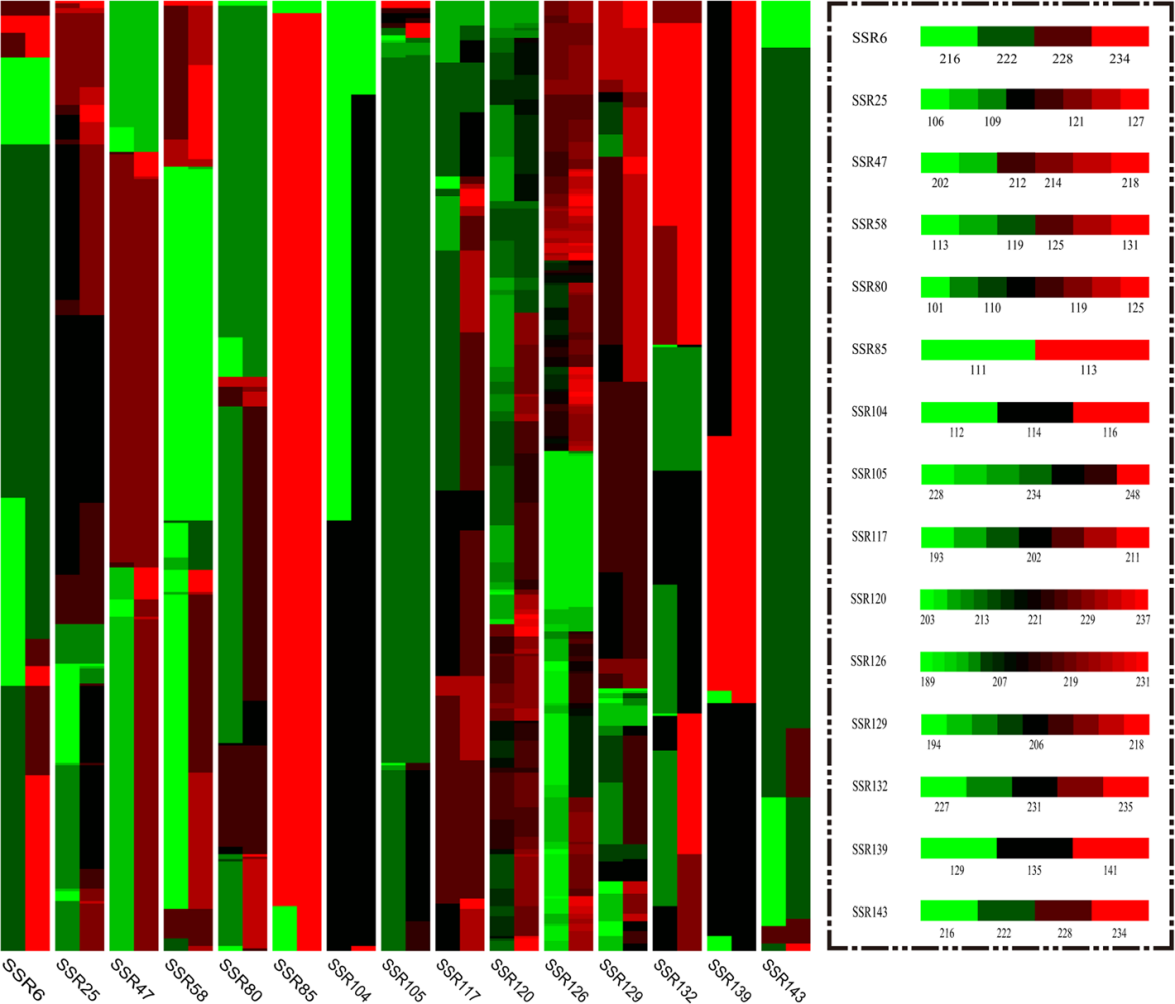
Heat map of the polymorphic SSR loci. The length of amplified fragments at each locus is represented by a colored band and each locus has a unique contrast diagram of fragment size and color. Different colors represent different fragment sizes, with greener colors indicating smaller fragments and redder colors indicating larger fragments

The heat map of polymorphic SSR loci revealed the genetic diversity information of the loci, with the color richness being related to the degree of polymorphism. Analysis of the results (Fig. 1) showed that loci SSR85, SSR104, and SR143 had monotone colors, indicating relatively poor levels of polymorphism, whereas other loci were rich in polymorphisms. In addition, homozygous and heterozygous information for the loci were also clearly and intuitively expressed in the heat map.

### Population genetic diversity

Based on the result of the population genetic diversity analysis (Additional file 1: Table S4), we found that the ‘Was’ population had relatively low genetic diversity (*N*_*a*_ = 2.60, *N*_*e*_ = 1.96, *I* = 0.617, *H*_*o*_ = 0.365, *H*_*e*_ = 0.358). The *I* values of the ‘Que’, ‘Lou’, and ‘Ten’ populations were 1.032, 1.131, and 1.175, respectively, and their genetic diversities were relatively high. Meanwhile these three populations had private alleles. The HWE results showed that the ‘Que’ and ‘Ten’ populations deviated significantly from the equilibrium.

**Fig. 2:**
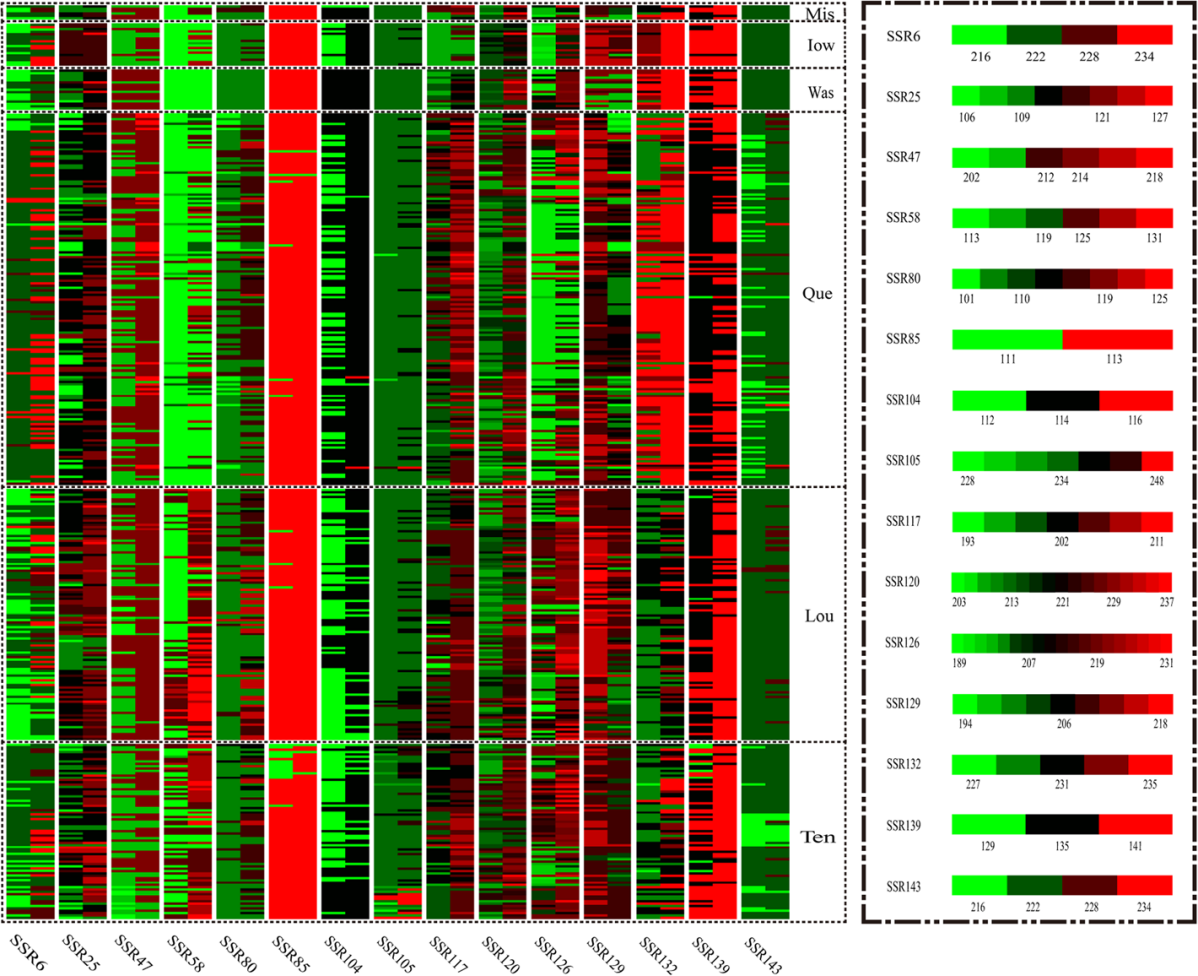
The heat map of population genetic diversity. The length of the amplified fragments at each locus is represented by a colored band and each locus has a unique contrast diagram of fragment size and color. Different colors represent different fragment sizes, a greener color indicates smaller fragments and a redder color indicates larger fragments. Individuals of the same population are surrounded by black dotted frames. ‘Mis’, ‘Iow’, ‘Was’, ‘Que’, ‘Lou’, ‘Ten’: different provenance populations respectively

The abundance of amplified fragments and the differences in the amplified fragments at each point in each population could be displayed visually using a heat map of the population genetic diversity (Fig. 2), which reflected the genetic variation of the population. The individuals from the ‘Was’ population were homozygous at the SSR58, SSR80, SSR85, SSR104, SSR105, and SSR143 loci, and its genetic diversity was poor. The size and proportion of amplified fragments could reveal the main genotypes and their differentiation in each population at each locus. Taking locus SSR143 as an example, single amplified fragments were obtained from the ‘Mis’, ‘Iow’, and ‘Was’ populations; some individuals in the ‘Que’ and ‘Ten’ populations were deleted on the basis of the main amplified fragments; and some individuals in the ‘Lou’ population produced amplicons with increased length.

### Population genetic differentiation and genetic variation

The *P. deltoides* population showed abundant genetic diversity and genetic differentiation. The within-population (*F*_*is*_) and inter-population (*F*_*it*_) inbreeding coefficients were used as indicators to evaluate the degree of population neatness. The inter-population genetic fraction coefficient (*F*_*st*_) was used as an indicator to evaluate the level of genetic differentiation of the populations. The existence of large gene flow (*N*_*m*_ > 1) among populations weakened the possibility of genetic drift, which would decrease the degree of genetic differentiation among populations. By contrast, when *N*_*m*_ < 1, the genetic differentiation among populations increases [32]. The average *F*_*is*_ and *F*_*it*_ values of *P. deltoides* were 0.058 and 0.182, respectively, indicating that there was a loss of heterozygosity in the population, and there was inbreeding among the populations. The *F*_*s*t_ between populations ranged from 0.062 to 0.205, with an average of 0.129, indicating that there was moderate genetic differentiation among the populations (Table 1). Meanwhile, higher gene flow (mean = 1.931, Table 1) among populations prevented genetic differentiation among populations to a certain extent.

**Table 1:** Genetic differentiation coefficients and gene flow of *P. deltoides* resources

Locus	*F*_*is*_	*F*_*it*_	*F*_*st*_	*N*_*m*_
SSR6	0.085	0.142	0.062	3.789
SSR25	−0.035	0.177	0.205	0.968
SSR47	0.199	0.302	0.128	1.703
SSR58	−0.149	0.067	0.188	1.083
SSR80	0.111	0.218	0.120	1.825
SSR85	0.280	0.328	0.066	3.535
SSR104	−0.067	0.077	0.135	1.608
SSR105	0.151	0.228	0.090	2.513
SSR117	−0.033	0.126	0.154	1.377
SSR120	−0.100	−0.003	0.088	2.586
SSR126	−0.016	0.111	0.125	1.752
SSR129	0.137	0.265	0.148	1.444
SSR132	−0.025	0.175	0.195	1.033
SSR139	−0.003	0.108	0.111	2.005
SSR143	0.328	0.413	0.126	1.740
Mean	0.058	0.182	0.129	1.931

*F*_*is*_: inbreeding coefficient within-population; *F*_*it*_: inbreeding coefficient inter-population; *F*_*st*_: inter-population genetic fraction coefficient; *N*_*m*_: gene flow

The result of analysis of molecular variance (AMOVA) showed that 84.88% of the total genetic variation originated from individuals, while 11.49% came from populations, and only 3.64% was ascribed to differences among individuals within the populations (Table 2). The results were consistent with the previous analysis results, which suggested that the genetic diversity of *P. deltoides* was mainly caused by genetic differences among individuals.

**Table 2:** Analysis of molecular variance (AMOVA) using 384 individuals from six populations of *P. deltoides* resources

Source of variation	*df*	Sum of squares	Variance of components	Percentage variation (%)	*P*-Value
Among populations	5	300.216	0.516	11.49	< 0.001
Among individuals within populations	378	1566.201	0.163	3.64	< 0.001
Within individuals	384	1465.500	3.816	84.88	< 0.001
Total	767	3331.917	4.496	100.00	

*df*: degrees of freedom

**Fig. 3:**
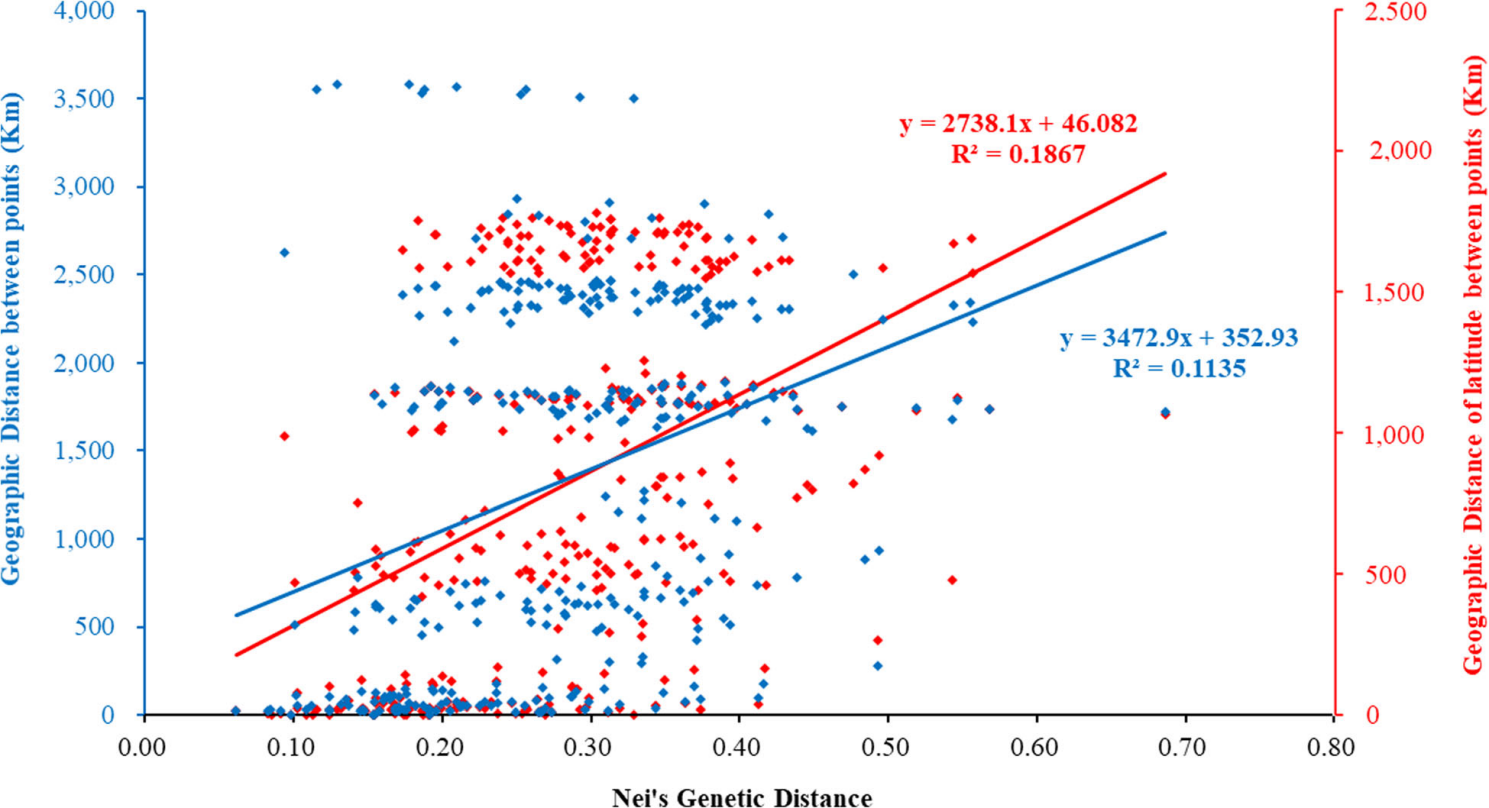
Correlation between Nei’s genetic distance and geographical distance (*P* < 0.01). The blue part represents the correlation between Nei’s genetic distance and geographical distance between points, and the red part represents the correlation between Nei’s genetic distance and latitude distance between points

Analysis using the Mantel test showed that there was a very significant relationship between genetic distance and geographical distance between points (R^2^ = 0.1135, *P* = 0.001, Fig. 3). Analysis of the correlation between the genetic distance and geographical distance of latitude, showed an extremely significant correlation and the fitting degree was relatively high (R^2^ = 0.1867, *P* = 0.001, Fig. 3).

### Genetic structure

**Fig. 4:**
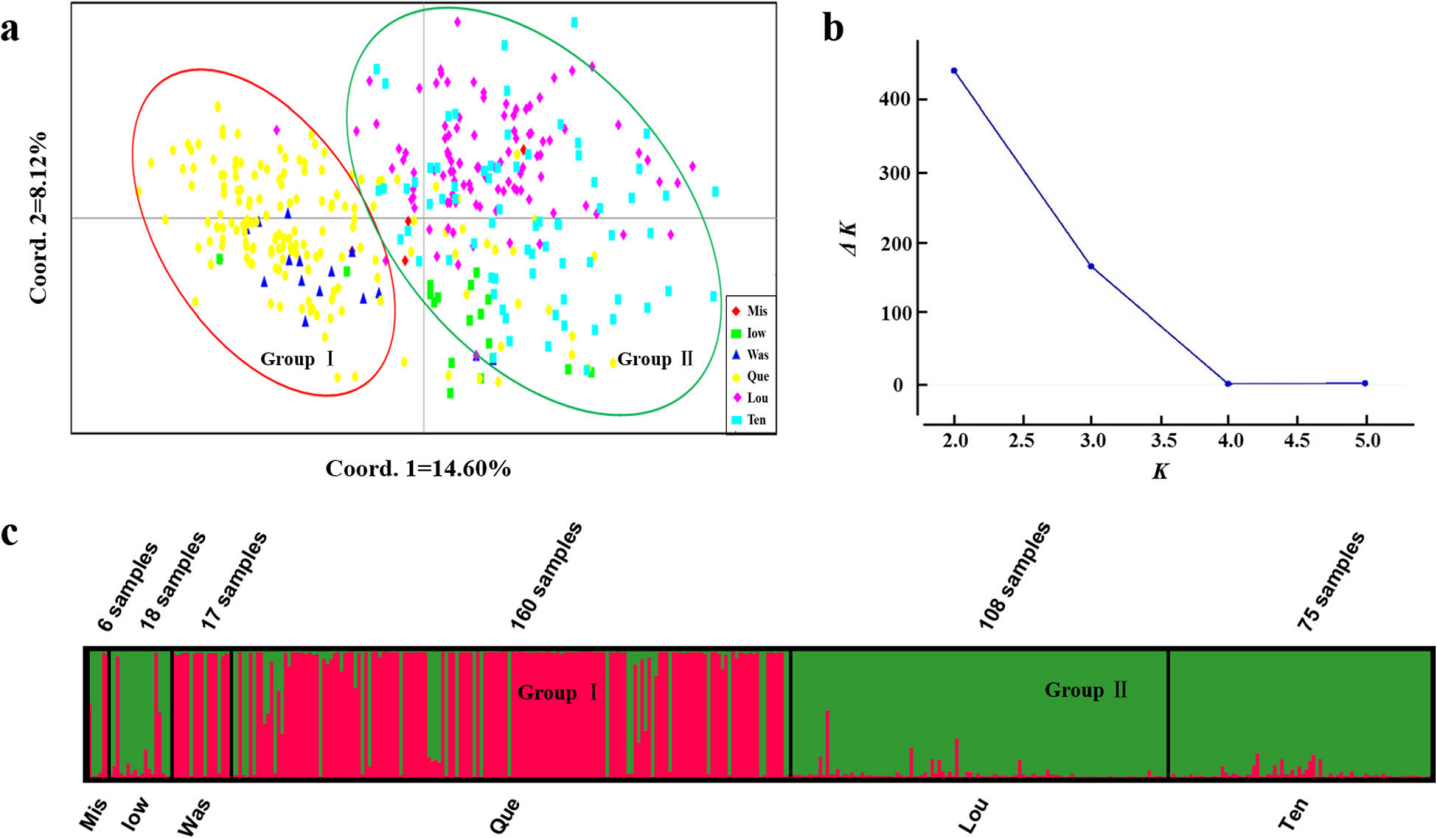
Analysis of the population structure of 384 *P. deltoides* Individuals. (**a**) Principal coordinates analysis (PCoA) of 384 individuals from six provenances. : the provenance population in Missouri, USA (‘Mis’); : the provenance population in Iowa, USA (‘Iow’); : the provenance population in Washington State, USA (‘Was’); : the provenance population in Quebec, Canada (‘Que’); : the provenance population in Louisiana, USA (‘Lou’); : the provenance population in Tennessee, USA (‘Ten’). Red circle area: Group I; Blue circle area: Group II. **(b)** Relations between the number of *K* and *ΔK*, based on the model developed by Evanno et al. [33]. **(c)** The population structure of *P. deltoides* determined using STRUCTURE 2.3.4 [34] software (*K* = 2). Red area: Group I; Blue area: Group II. ‘Mis’, ‘Iow’, ‘Was’, ‘Que’, ‘Lou’, ‘Ten’: different provenance populations respectively

The results of principal coordinate analysis (PCoA) showed that the 384 individuals of *P. deltoides* could be divided into two groups (Fig. 4a). Group I mainly included the individuals from ‘Que’ and ‘Was’, and a few individuals from the ‘Iow’ provenance. Group II mainly included the individuals of the ‘Ten’, ‘Lou’, ‘Mis,’ and ‘Iow’ provenances. The individuals from the ‘Que’ provenance were widely distributed, which indicated that ‘Que’ had abundant genetic diversity. STRUCTURE cluster analysis showed that when *K* = 2, the *ΔK* value was relatively large (Fig. 4b), which indicated that the 384 *P. deltoides* individuals could be divided into two different groups. The genetic structure map (Fig. 4c) showed that most individuals from ‘Que’ and ‘Was’ belonged to the red group (Group I), while most individuals from ‘Ten’, ‘Lou’, and ‘Iow’ belonged to the green group (Group II). Based on the analysis of the matrix of its estimated membership probability (Q-matrix) when *K* = 2, we could determine the composition of the red and green groups (Additional file 1: Table S5).

**Fig. 5:**
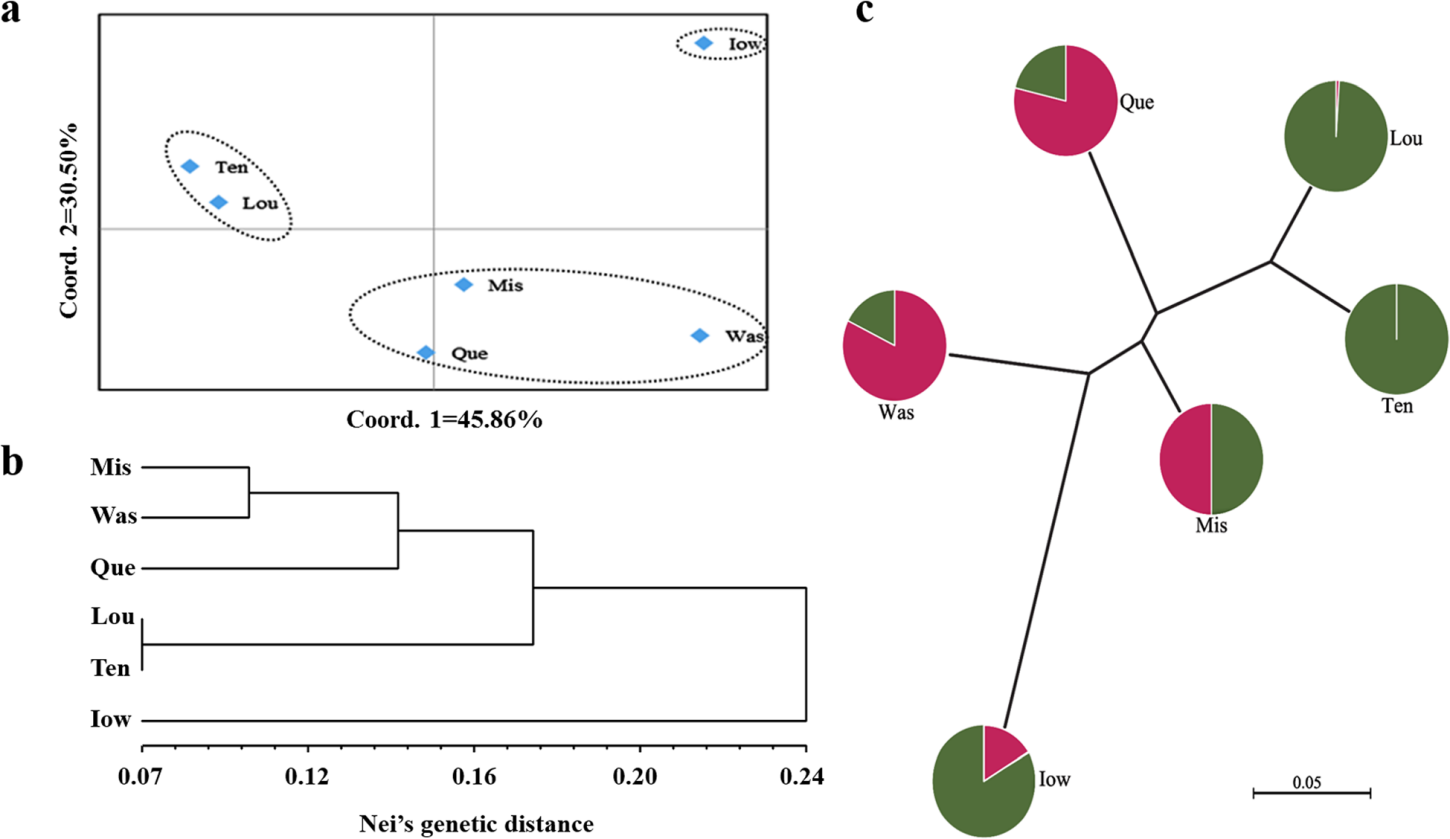
Population structure of six provenance populations of *P. deltoides*. (**a**) The principal coordinates analysis (PCoA) of six populations of *P. deltoides*. (**b**) UPGMA analysis of six populations of *P. deltoides* based on Nei’s genetic distance. **(c)** The unrooted tree based on Nei’s genetic distance for six *P. deltoides* populations. The pie chart reflects the distribution proportion of individuals of the provenance populations in the two groups

To understand the genetic structure among the six populations of *P. deltoides*, we carried out PCoA and cluster analysis between the populations, based on the unweighted pair-group method with arithmetic means (UPGMA) on the provenance populations (Fig. 5a, b). In addition, an unrooted tree was drawn (Fig. 5c). The results showed that the ‘Lou’ and ‘Ten’ provenances were clustered together; the ‘Mis’, ‘Was,’ and ‘Que’ provenances were clustered together; and ‘Iow’ was independent of the other provenance populations.

## Discussion

### Null alleles

SSR molecular markers have been widely used to study population genetic diversity and genetic structure. However, the existence of null alleles might reduce the population genetic diversity and increase genetic differentiation among populations, which will have a significant impact on the results of the study [35]. In this study, five pairs of primers with a high frequency of null alleles were identified. To clearly understand the effect of loci containing null alleles on the population genetic analysis results, we compared the changes of major genetic parameters before and after deletion of these loci. We found the difference between the *H*_*o*_ and *H*_*e*_ values increased, the *F*_*st*_ increased, and the *N*_*m*_ decreased in the presence of the null allele loci (Table 3). These changes indicated that the five loci had null alleles that likely contributed to the positive genetic fraction coefficient, and affected the interpretation of the results [36].

**Table 3:** Statistical table of genetic diversity parameters with and without null allele loci

	*Ho* (pop.)	*He* (pop.)	*F*_*st*_	*N*_*m*_
Delete null allele loci	0.466	0.487	0.129	1.931
Include null allele loci	0.444	0.515	0.131	1.859

*Ho* (pop.): mean of observed heterozygosity of population; *He* (pop.): mean of expected heterozygosity of population; *F*_*st*_: inter-population genetic fraction coefficient; *N*_*m*_: gene flow

### SSR primer screening

**Fig. 6:**
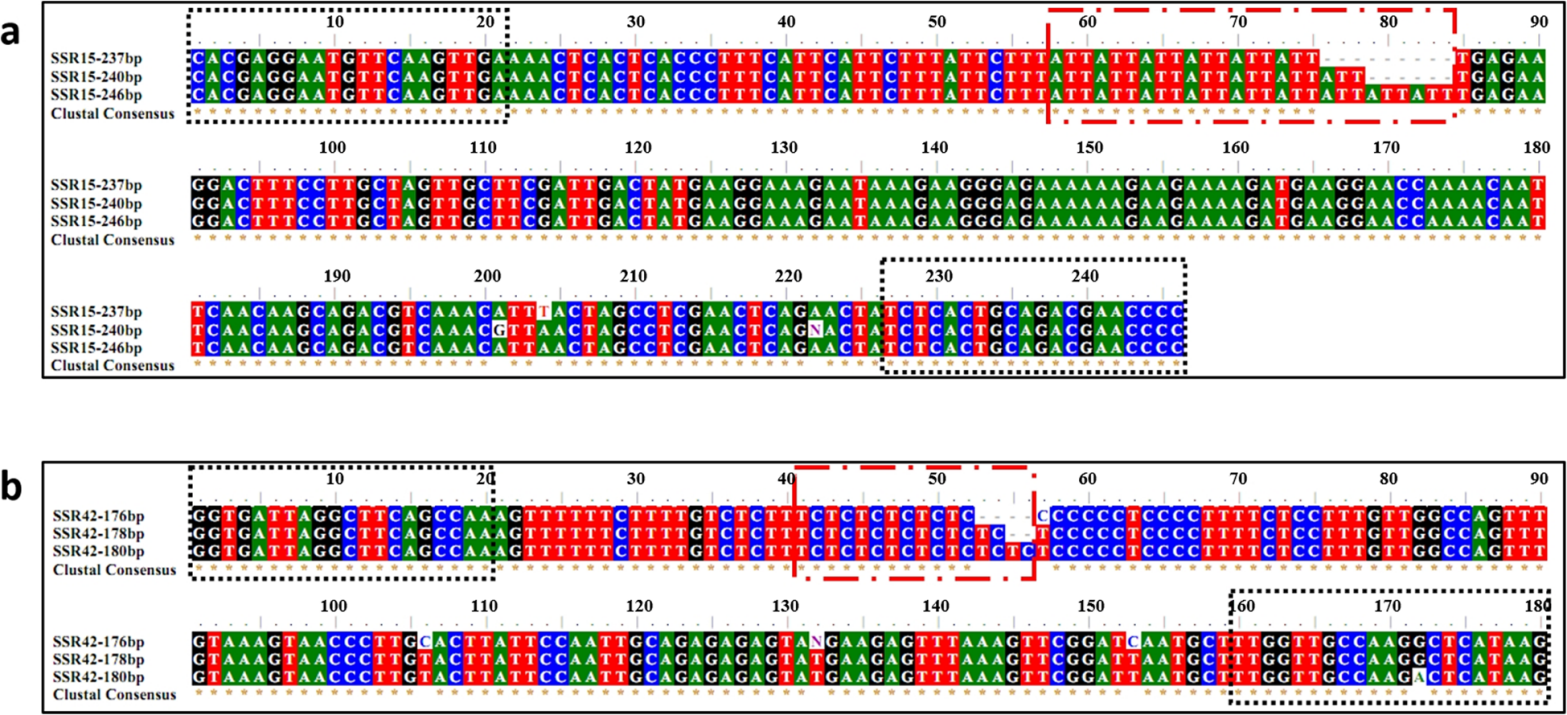
Amplification sequence alignment diagram of SSR15 (**a**) and SSR42 (**b**). The base sequence in the black dotted frame matches the corresponding primer sequence, the sequence in the red dotted frame is a simple repeat sequence

The SSR primers based on functional gene sequence analysis of poplar have different characteristics in different varieties. In this study the amplification motifs of two primer pairs, SSR15 and SSR42, showed some differences. The SSR15 locus was located in the promoter region of the *PsHsf16* gene of the *P. simonii* Hsf family. In a previous study [23], its repeat unit was ATTT; however, in this study, the repeat motif was ATT (Fig. 6a). The SSR42 locus was in the intron region of the *PtCesA6* gene in *P. tomentosa*, and its repeating motif was TTCTCC [25], whereas, in this study, the amplifying motif was TC (Fig. 6b). Meanwhile, we observed that the molecular markers of the functional genes of other poplar varieties screened in this study were likely to generate null alleles. These results indicated that the marker characteristics of the same primers might change when they are applied to different experimental materials [37]. Therefore, the stability and polymorphism of primers should be further verified when screening primers that have been developed based on the analysis of functional gene sequences. Of course, it would be better to develop species-specific SSR markers, which is conducive to further research.

### Genetic diversity and variation

A better understanding of the genetic diversity, genetic variation, and genetic regulation of a population is essential for its proper management and conservation, especially in the face of current climate change, and the genetic evaluation of groups such as forests is particularly important [38]. Fahrenkrog et al. performed the first population genomics study for 425 unrelated individuals of *P. deltoides* distributed in 13 states of the southeastern United States. By assessing population structure, population differentiation, genetic diversity and adaptation in *P. deltoides*, they found that the differentiation between subpopulations of the natural *P. deltoides* population was weak (*F*_*st*_ = 0.022–0.106); however, the genetic diversity was high [29]. Furthermore, genome-wide association studies (GWASs) were also used to dissect the genetic regulation of eight growth and wood composition traits in *P. deltoides*, and single-nucleotide polymorphisms were detected by targeted resequencing of 18,153 genes in a population of 391 unrelated individuals. The authors found that both common and low frequency variants need to be considered to understand the genetic regulation of complex traits [39]. In the present study, the genetic diversity and population structure of 384 individuals of *P. deltoides* from six provenances in the Saint Lawrence River Basin (Quebec, Canada), Columbia River Basin (Washington, USA) and Mississippi River Basin (Missouri, Iowa, Tennessee and Louisiana, USA) were analyzed using the SSR marker technique. The results showed that the *P. deltoides* had abundant genetic diversity and moderate genetic differentiation*.* In addition, the degree of genetic differentiation was relatively high, probably because the materials in this study were from three basins in the main distribution area of *P. deltoides*, and the materials in Fahrenkrog’s study were mainly from the Mississippi River Basin [29, 39]. Our research also showed that geographical isolation hindered gene exchange among individuals from different provenances and aggravated genetic differentiation. In addition, the genetic distance between the provenances in the north (‘Que’, ‘Was’) and the south (‘Ten’, ‘Lou’) was relatively large, while the genetic distance between the provenances in the east (‘Que’) and the west (‘Was’) was relatively small. The reason for this result was consistent with the climate distribution in North America.

### Population structure

In this study, we analyzed the population structure of *P. deltoides* at different levels. At the individual level, PCoA and STRUCTURE cluster analysis were performed on 384 unrelated individuals, which divided them into two groups. Most of the individuals distributed in the Mississippi River Basin were grouped together (Group II, Fig. 4a, c), and individuals in different states of the Mississippi River Basin had high genetic similarity, which was consistent with the results of Fahrenkrog et al. [29, 39]. The genetic differentiation between different basins indicated that geographical isolation restricts gene exchange among populations [40]. Individuals distributed in the Saint Lawrence River Basin and the Columbia River Basin were grouped into the other group (Group I, Fig. 4a, c). These two locations might have the same type of genetic variation because of their similar environment and latitudes. Taking the provenances as the research object, the ‘Lou’ and ‘Ten’ provenances were clustered into one group, the ‘Mis’, ‘Was’ and ‘Que’ provenances were clustered into another group, and the ‘Iow’ provenance formed a third group (Fig. 5). To determine the reasons for the differences between the results of individuals and provenances analysis, we analyzed *N*_*m*_ and *F*_*st*_ among the six provenances (Table 4). We observed that the *N*_*m*_ between ‘Iow’ and the others was relatively small and the *F*_*st*_ was large, which could explain why the ‘Iow’ provenance was separately classified into one group [41]. Eighteen individuals from ‘Iow’ were selected, and PCoA of 384 individuals of *P. deltoides* showed that they were distributed widely in the dimension of the first principal component, and most individuals were on the edge of Group II (Fig. 4a). We hypothesized that individuals from ‘Iow’ might have been subjected to some external selection pressure, which has resulted in genetic variation in a certain direction. This selection pressure also exists in other provenances; however, the selection pressure is relatively weak and did not lead to directional mutations of genes. Therefore, when analyzing population structure, we should not neglect the influence of external environmental conditions on the structure. At the same time, geographical isolation has restricted gene exchange between ‘Iow’ and other provenances.

**Table 4:** Gene flow (*N*_*m*_, above diagonal) and genetic differentiation coefficient (*F*_*st*_, below diagonal) between the six provenances

	Mis	Iow	Was	Que	Lou	Ten
Mis	–	1.885	2.959	4.476	4.476	3.681
Iow	0.117	–	1.618	2.038	2.155	2.238
Was	0.078	0.134	–	2.666	2.056	1.785
Que	0.053	0.109	0.086	–	4.469	4.250
Lou	0.049	0.104	0.108	0.053	–	8.469
Ten	0.064	0.100	0.123	0.056	0.029	–

‘Mis’, ‘Iow’, ‘Was’, ‘Que’, ‘Lou’, ‘Ten’: different provenance populations respectively. *F*_*st*_: inter-population genetic fraction coefficient; *N*_*m*_: gene flow

### Management and utilization of Germplasm resources

The management of germplasm resources is a complex task. We should have a full understanding of their genetic information, morphological diversity, and adaptability. Meanwhile, germplasm resources need to be effectively identified to prevent redundancy between resources [42, 43]. Germplasm banks play an important role in the conservation, management, and utilization of germplasm resources that is critical for the development of plant breeding [44]. Molecular genetic markers are widely used in germplasm identification, and they are important in the construction and management of germplasm banks [45]. For example, the single nucleotide polymorphism (SNP) markers were used to identify *Dimocarpus longan* L. germplasm [46] and *Discorea alata* L. germplasm [47]. Reyes-Valdés et al. offered an integrated view of accession rarity and allele specificity in germplasm banks for management and conservation [45]. Storme et al. [48] analyzed 675 *P. nigra* L. accessions from nine European gene banks using SSR markers, amplified fragment length polymorphism (AFLP) markers and isozyme systems to estimate the extent of duplication and the genetic diversity within and between banks. To better manage and utilize *P. tomentosa* [20] and *P. simonii* [49] resources, their genetic diversity had been studied using SSR markers. Combined with the analysis of phenotypic diversity of *P. deltoides*, we may identify the key polymorphic loci associated with the traits through correlation analysis, which is conducive to the development of molecular marker-assisted breeding or detection of target genes in the near future. In addition, a core germplasm bank could be built to rationally manage, preserve, and utilize the *P. deltoides* resources.

## Conclusions

In the present study, the genetic diversity and population structure of *P. deltoides* germplasm resources were analyzed. The results showed that they had abundant genetic diversity. However, there were also some differences in genetic diversity among different provenances, with moderate genetic differentiation (mean *F*_*st*_ = 0.129, Table 1). The genetic variation mainly came from individuals in different provenances. Geographical isolation was the main reason for the differences among the provenances. Based on the results, representative individuals could be selected to form a core germplasm bank of *P. deltoides* to improve the selection efficiency of hybrid parents of poplar and to lay a scientific foundation for the conservation and breeding of poplar germplasm resources.

## Methods

### Plant materials and DNA extraction

**Fig. 7:**
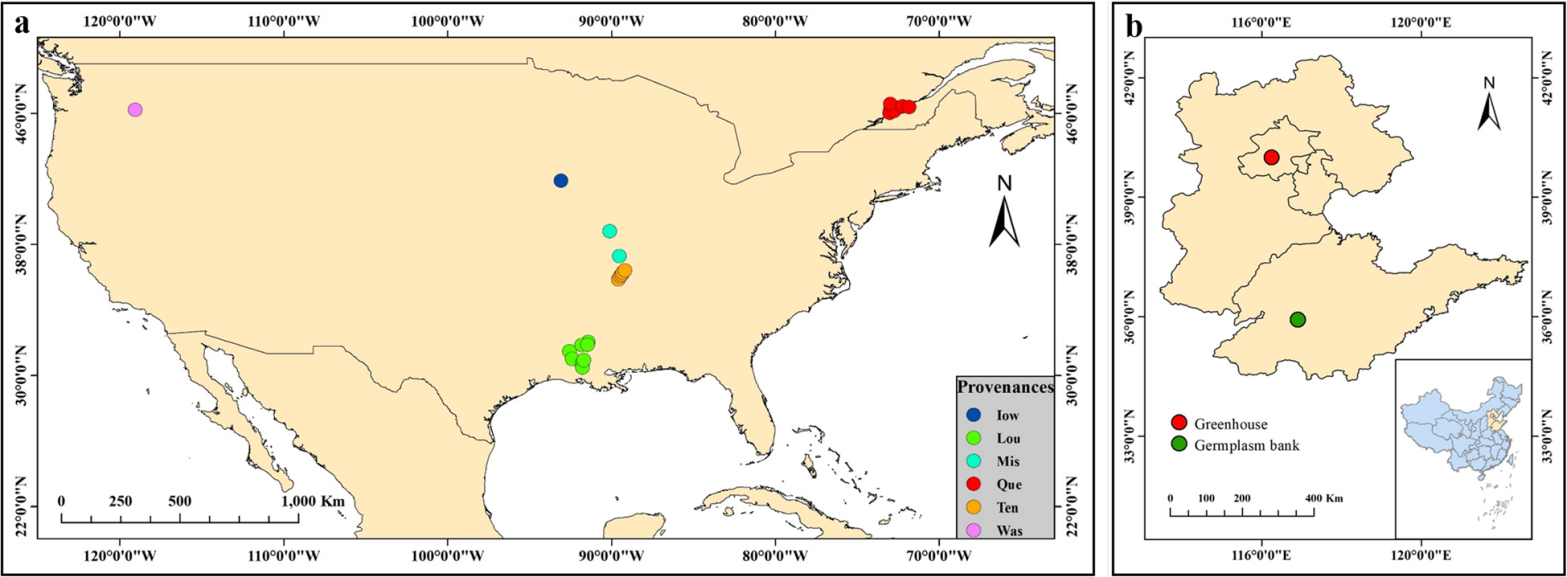
Geographical distribution of provenances (**a**) and the germplasm bank (**b**) of *P. deltoides*

Cuttings of *P. deltoides* were collected from the germplasm bank (35°55′39″N, 116°53′59″E) that was established through international exchange and cooperation of germplasm resources in 2009, among which the resources came from its main distribution areas. The materials have been formally identified by the State Forestry and Grassland Administration, People’s Republic of China, under the identification number 2009–56. In this study, 384 unrelated individuals were randomly selected from six provenances. When collecting the materials, several collection sites were selected in each provenance, and then individual materials were collected near the collection sites. The geographical distance between individuals was more than 100 m. Six individuals were selected from two sampling sites in Missouri (‘Mis’). Eight sampling sites were selected in Louisiana (‘Lou’) and five in Tennessee (‘Ten’); 108 individuals and 75 individuals were selected, respectively. Eighteen unrelated individuals were from Iowa (‘Iow’). The four provenances were located in Mississippi River basin. Seventeen unrelated individuals were from Washington (‘Was’), which is located in the Columbia River basin. In addition, 160 unrelated individuals were from ten sampling sites in the Saint Lawrence River basin (Quebec, Canada, ‘Que’) (Fig. 7, Additional file 1: Table S6).

The plant materials were cultured in the greenhouse of the Chinese Academy of Forestry (40°0′10″N 116°14′38″E), and the leaves were collected during the growing season and stored in a refrigerator at − 40 °C for DNA extraction. Total genomic DNA was extracted according to the highly efficient modified cetyltrimethylammonium bromide (CTAB) method [50]. The quality and integrity of the extracted genomic DNA were assessed using 1% agarose gel electrophoresis. The DNA concentration was then determined using a NanoDrop-2000 ultramicro-spectrophotometer (Thermo Fisher Scientific Waltham, MA, USA). Finally, the DNA was diluted to 50 ng·μL^− 1^ and stored at − 20 °C for polymerase chain reaction (PCR) amplification.

### Primer selection and PCR amplification

In this study, DNA of 20 unrelated individuals *P. deltoides* were selected randomly for SSR primer screening, and 145 pairs of primers were used for screening. Seventy-five of the primer pairs were developed by researchers based on the analysis of functional gene sequences in poplar [23–27, 51]. Another 70 pairs of primers were obtained from the International Populus Genome Consortium (IPGC, http://www.ornl.gov/sci/ipgc/ssr_resource.htm) and Washington University (Poplar Molecular Genetics Cooperative, http://poplar2.cfr.washington.edu), which have used by other researchers in related studies [49, 52–54]. The stability and polymorphism of the SSR primers were preliminarily screened by PCR amplification, 2% agarose gel electrophoresis, and 8% non-denaturing polyacrylamide gel electrophoresis.

The PCR amplification reaction system for all SSR markers comprised a 25 μL mixed system containing 2.5 μL of 10 × buffer (Mg^2+^ plus), 1.8 μL of dNTP mixture, 1 μL of forward primer (10 μmol·L^− 1^), 1 μL of reverse primer (10 μmol·L^− 1^), 0.25 μL of *Taq* polymerase (5 U·μL^− 1^), 1 μL of DNA template (50 ng·μL^− 1^), and 17.45 μL of ddH_2_O. The PCR amplification procedure comprised 94 °C for 3 min; 35 cycles of 94 °C for 30 s, annealing at the annealing temperature of each primer pair for 30 s, elongation at 72 °C for 45 s; and a final extension at 72 °C for 10 min.

The forward primers for the preliminarily screened polymorphic loci were labeled with fluorescent dyes (5-HEX or 5-FAM) and their PCR products were separated by capillary electrophoresis using an ABI 3730xl DNA analyzer (Applied Biosystems, Foster City, CA, USA). A peak size map of the amplified fragments was obtained.

### Data analysis

The Gene-Marker 2.2.0 software (SoftGenetics LLC, USA) was used to read the peak maps of the amplified fragments of each polymorphic locus. The null allele frequencies of each locus were detected using Cervus 3.0.7 [30] and Microchecker 2.2.3 [31] software, and the common detection results were taken. The Cervus 3.0.7 software was also used to calculate the *PIC* of each locus.

The GeneAlEx 6.503 [55] software was used to convert various file formats for different analysis and to calculate genetic diversity parameters, including the number of alleles (*N*_*a*_), the effective number of alleles (*N*_*e*_), Shannon’s information index (*I*), observed heterozygosity (*H*_*o*_), expected heterozygosity (*H*_*e*_), the number of private alleles, gene flow (*N*_*m*_), and the *F*-Statistics (*F*_*is*_*, F*_*it*_*, F*_*st*_). The HWE tests across all provenances were performed using Genepop 4.7.0 [56]. The heat maps of each locus were drawn using OmicShare tools (www.omicshare.com/tools). With the help of the Arlequin 3.5.2.2 [57] and GeneAlEx 6.503 software, AMOVA was carried out to partition the genetic variances into three levels: Among populations, among individuals within populations, and within individuals.

We further calculated the genetic distance between individuals and populations using the GeneAlEx 6.503 software for PCoA. A cluster analysis between populations, based on UPGMA, was also developed using the NTSYS-pc 2.10e [58] software. An unrooted tree was constructed based on pairwise standard genetic distances [59], using the least squares algorithm with 10,000 bootstrap replicates, and these processes were generated and analyzed using PHYLIP 3.6 [60] software.

The population genetic structure was analyzed using STRUCTURE 2.3.4 [34] software, using a model-based clustering algorithm that implements a Bayesian framework and the Markov chain Monte Carlo (MCMC) algorithm. To confirm the optimum number of subpopulations (*K*), 10 independent runs for each value of *K*, ranging from 2 to 5, were conducted. Each run consisted of a burn-in period of 100,000 steps followed by 1000,000 MCMC iterations. The *ΔK* parameter, which was based on the rate of change in the log probability of data between successive *K* values, was estimated to determine the best *K*, based on the model developed by Evanno et al. [33].

The Mantel test was performed using the GeneAlEx 6.503 software to analyze the correlation between Nei’s genetic distance and geographical distance. The geographical distance between different sites was calculated according to the latitude and longitude using Vincenty’s formula (www.movable-type.co.uk/scripts/latlong-vincenty.html).

## Supplementary information

**Additional file 1: Table S1.** Information for the SSR primers. **Table S2.** The detection of null allele loci using SSR primers based on two analyses software. **Table S3.** The genetic diversity parameters of 15 SSR primers in *P. deltoids* resources. **Table S4.** Genetic diversities of the six populations of *P. deltoides* resources. **Table S5.** Composition of the members of two groups according to Q-value matrix (*K* = 2). **Table S6.** Material Information for *P. deltoides* germplasm resources. **Table S7.** Genetic information of polymorphic loci.

## Data Availability

All data generated or analyzed during this study are included in this published article [and its additional files]. Genetic information of polymorphic loci has been uploaded as Additional file 1: Table S7.

## Abbreviations

‘Iow’: The provenance population in Iowa, USA
‘Lou’: The provenance population in Louisiana, USA
‘Mis’: The provenance population in Missouri, USA
‘Que’: The provenance population in Quebec, Canada
‘Ten’: The provenance population in Tennessee, USA
‘Was’: The provenance population in Washington State, USA.
AMOVA: Analysis of molecular variance
*F*_*is*_: Inbreeding coefficient within-population
*F*_*it*_: Inbreeding coefficient inter-population
*F*_*st*_: Inter-population genetic fraction coefficient
HWE: Hardy-Weinberg equilibrium
*N*_*m*_: Gene flow
PCoA: Principal coordinate analysis
*PIC*: Polymorphic information content
SSR: Simple sequence repeat
UPGMA: Unweighted pair-group method with arithmetic means
