# Supplementary material for: Genetic diversity and population structure of black cottonwood (Populus deltoides) revealed using simple sequence repeat markers
Source: BMC Genet. 2020 Jan 6;21:2. doi: 10.1186/s12863-019-0805-1 (PMC6945526; doi:10.1186/s12863-019-0805-1)
Supplement: Supplementary file 1 — Additional file 1: Table S1. Information for the SSR primers. Table S2. The detection of null allele loci using SSR primers based on two analyses software. Table S3. The genetic diversity parameters of 15 SSR primers in P. deltoids resources. Table S4. Genetic diversities of the six populations of P. deltoides resources. Table S5. Composition of the members of two groups according to Q-value matrix (K = 2). Table S6. Material Information for P. deltoides germplasm resources. Table S7. Genetic information of polymorphic loci. [file 12863_2019_805_MOESM1_ESM.zip › Table S4.docx]

**Table S4.** **Genetic diversities of the six populations of *P. deltoides* resources**

| **Population** | **Sample**  **number** | ***N_a_*** | ***N_e_*** | ***I*** | ***H_o_*** | ***H_e_*** | **Number of**  **private alleles** | **HWE(*P*)** |
| --- | --- | --- | --- | --- | --- | --- | --- | --- |
| Mis | 6 | 3.13 | 2.55 | 0.833 | 0.389 | 0.502 | 0 | 0.011 |
| Iow | 18 | 3.07 | 1.98 | 0.675 | 0.456 | 0.390 | 0 | 0.995 |
| Was | 17 | 2.60 | 1.96 | 0.617 | 0.365 | 0.358 | 0 | 0.465 |
| Que | 160 | 5.93 | 2.66 | 1.032 | 0.475 | 0.511 | 11 | 0.000^*^ |
| Lou | 108 | 5.73 | 3.30 | 1.131 | 0.578 | 0.565 | 6 | 0.484 |
| Ten | 75 | 5.87 | 3.10 | 1.175 | 0.536 | 0.595 | 3 | 0.000^*^ |
| Mean | -- | 4.39 | 2.59 | 0.910 | 0.466 | 0.487 | -- | -- |

‘Mis’, ‘Iow’, ‘Was’, ‘Que’, ‘Lou’, ‘Ten’: different provenance populations respectively. *N_a_*: number of alleles per locus; *N_e_*: effective number of alleles; *I*: Shannon’s information index; *H_o_*: observed heterozygosity; *H_e_*: expected heterozygosity; HWE: Hardy-Weinberg equilibrium; * indicates significant deviations from Hardy-Weinberg equilibrium (Bonferroni adjusted P value<0.05/15=0.0033).
